# Supplementary material for: The poly-SUMO2/3 protease SENP6 enables assembly of the constitutive centromere-associated network by group deSUMOylation
Source: Nat Commun. 2019 Sep 4;10:3987. doi: 10.1038/s41467-019-11773-x (PMC6726658; doi:10.1038/s41467-019-11773-x)
Supplement: Supplementary file 1 — Supplementary Information [file 41467_2019_11773_MOESM1_ESM.pdf]

Supplementary Information

The poly-SUMO2/3 protease SENP6 enables assembly of the constitutive centromere-associated network by group deSUMOylation

Liebelt et al.

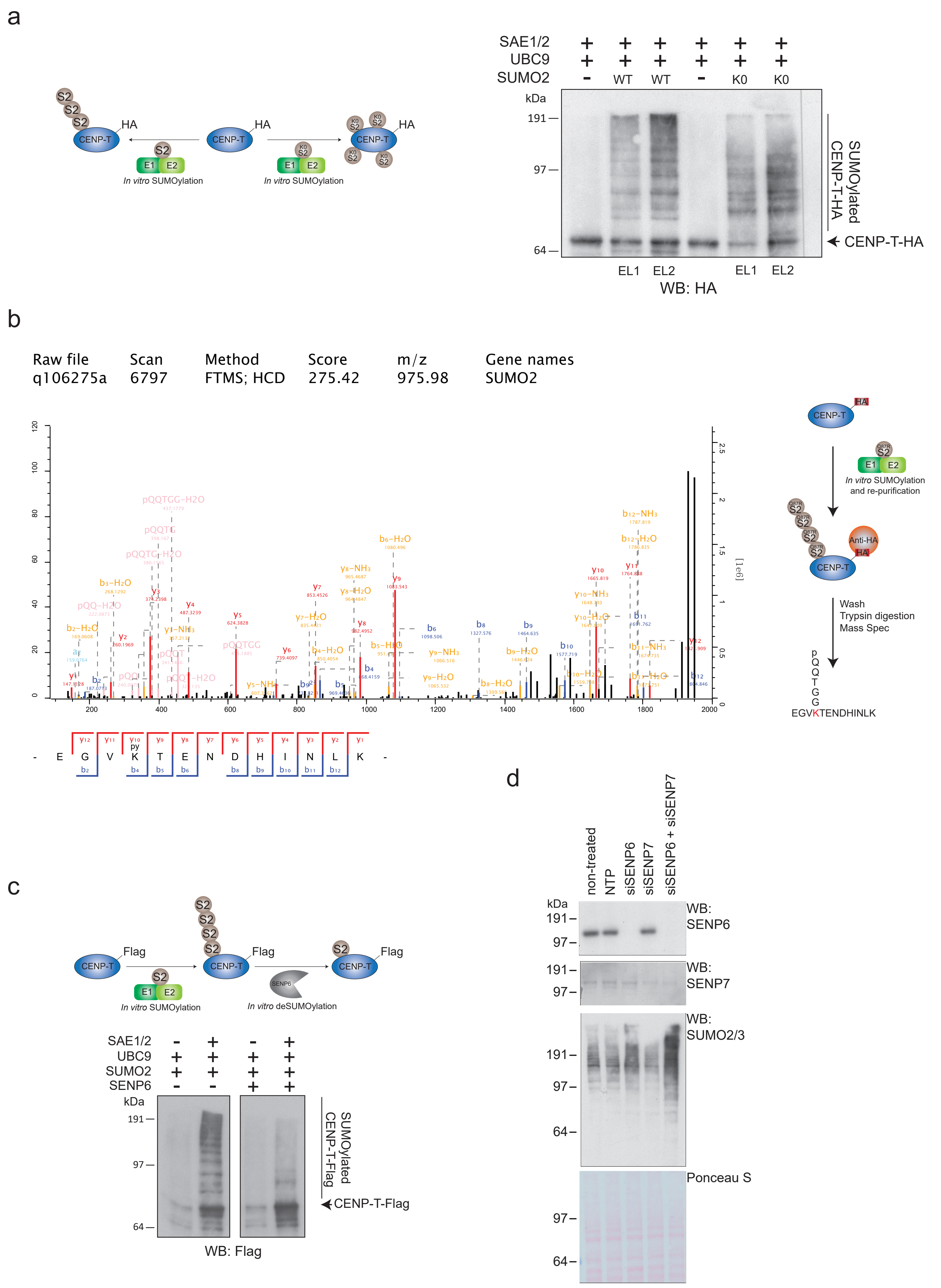
a
SAE1/2
UBC9
SUMO2
WT
K0
kDa
191
97
64
SUMOylated CENP-T-HA
CENP-T-HA
EL1
EL2
WB: HA
CENP-T
HA
S2
E1
E2
In vitro SUMOylation
b
Raw file
q106275a
Scan
6797
Method
FTMS; HCD
Score
275.42
m/z
975.98
Gene names
SUMO2
E G V K T E N D H I N L K
In vitro SUMOylation and re-purification
Anti-HA
Wash
Trypsin digestion
Mass Spec
EGVKTENDHINLK
c
Flag
SENP6
In vitro deSUMOylation
SAE1/2
UBC9
SUMO2
SENP6
kDa
191
97
64
SUMOylated CENP-T-Flag
CENP-T-Flag
WB: Flag
d
non-treated
NTP
siSENP6
siSENP7
siSENP6 + siSENP7
kDa
WB: SENP6
WB: SENP7
WB: SUMO2/3
Ponceau S

Supplementary Fig. 1 SENP6 deSUMOylates poly-SUMO2/3 modified CENP-T.

a CENP-T-HA was purified from cells and eluted fractions one and two (EL1 and EL2) were *in vitro* SUMOylated with either recombinant SUMO2 WT or lysine-deficient SUMO2 (K0). CENP-T-HA was visualized by immunoblotting, using an antibody against the HA epitope. Unmodified CENP-T-HA is indicated by an arrow. Modification with SUMO2 WT but not with SUMO2 K0 led to high-molecular weight conjugates of CENP-T, indicating the presence of SUMO chains.

b Purified CENP-T-HA was *in vitro* SUMOylated with recombinant SUMO2 Q87R, re-purified, washed and trypsin digested. MS/MS spectrum representing (pyro)QQTGG modified K11-SUMO peptide identified by mass spectrometry. The presence of the branched peptide SUMO K11 - (pyro)QQTGG on *in vitro* SUMOylated CENP-T indicates the presence of SUMO chains on CENP-T.

c *In vitro* SUMOylated CENP-T-Flag was treated with recombinant SENP6. CENP-T-Flag was visualized by immunoblotting, using an antibody against the Flag epitope. Unmodified CENP-T-Flag is indicated by an arrow. Recombinant SENP6 was able to efficiently deSUMOylate *in vitro* SUMOylated CENP-T.

d U2OS cells were left untreated or transfected with either a pool of four siRNAs against SENP6 (siSENP6), SENP7 (siSENP7), a combination of both (siSENP6 + siSENP7) or a pool of four non-targeting siRNAs (NTP). Cell lysates were analysed two days post transfection by immunoblotting, using antibodies against SENP6, SENP7 and SUMO2/3. Source data are provided as a Source Data file.

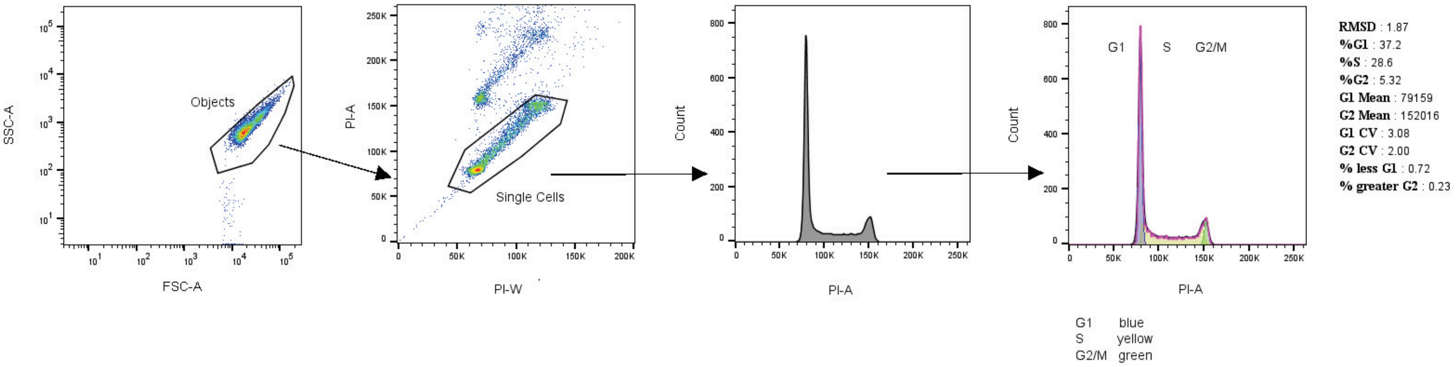

Objects
SSC-A
FSC-A
Single Cells
PI-A
PI-W
Count
PI-A
G1
S
G2/M
Count
PI-A
RMSD : 1.87
%G1 : 37.2
%S : 28.6
%G2 : 5.32
G1 Mean : 79159
G2 Mean : 152016
G1 CV : 3.08
G2 CV : 2.00
% less G1 : 0.72
% greater G2 : 0.23
G1 blue
S yellow
G2/M green

Supplementary Fig. 2 Gating strategy for cell cycle analysis upon SENP6 knockdown.

Gating strategy underlying Fig. 1e. HeLa cells were treated with lentiviruses encoding shRNAs against SENP6 or a non-targeting control shRNA. Cells were fixed and prepared for flow cytometry analysis four days post infection.

a

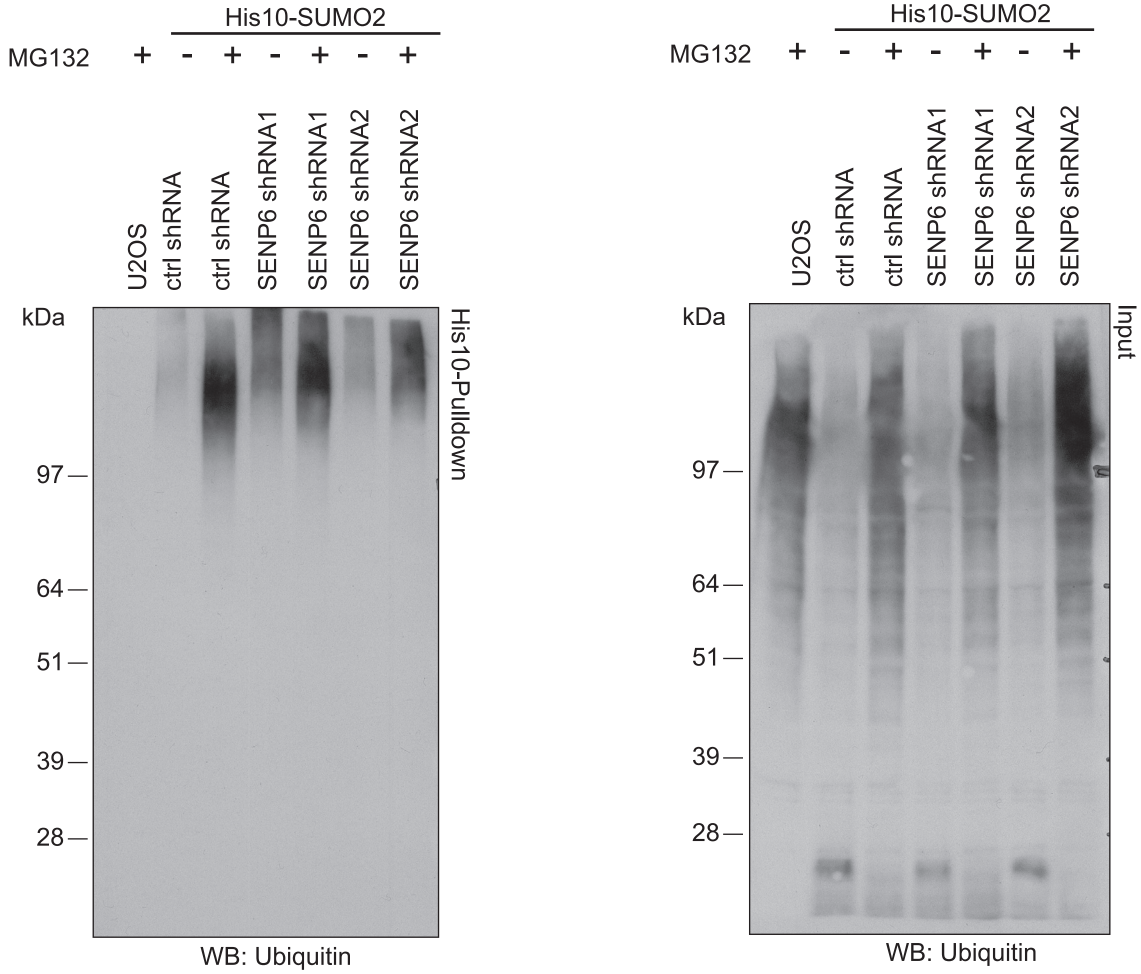

His10-SUMO2
MG132
+ - + - + - +
U2OS
ctrl shRNA
ctrl shRNA
SENP6 shRNA1
SENP6 shRNA1
SENP6 shRNA2
SENP6 shRNA2
kDa
97
64
51
39
28
His10-Pulldown
WB: Ubiquitin
His10-SUMO2
MG132
+ - + - + - +
U2OS
ctrl shRNA
ctrl shRNA
SENP6 shRNA1
SENP6 shRNA1
SENP6 shRNA2
SENP6 shRNA2
kDa
97
64
51
39
28
Input
WB: Ubiquitin

b

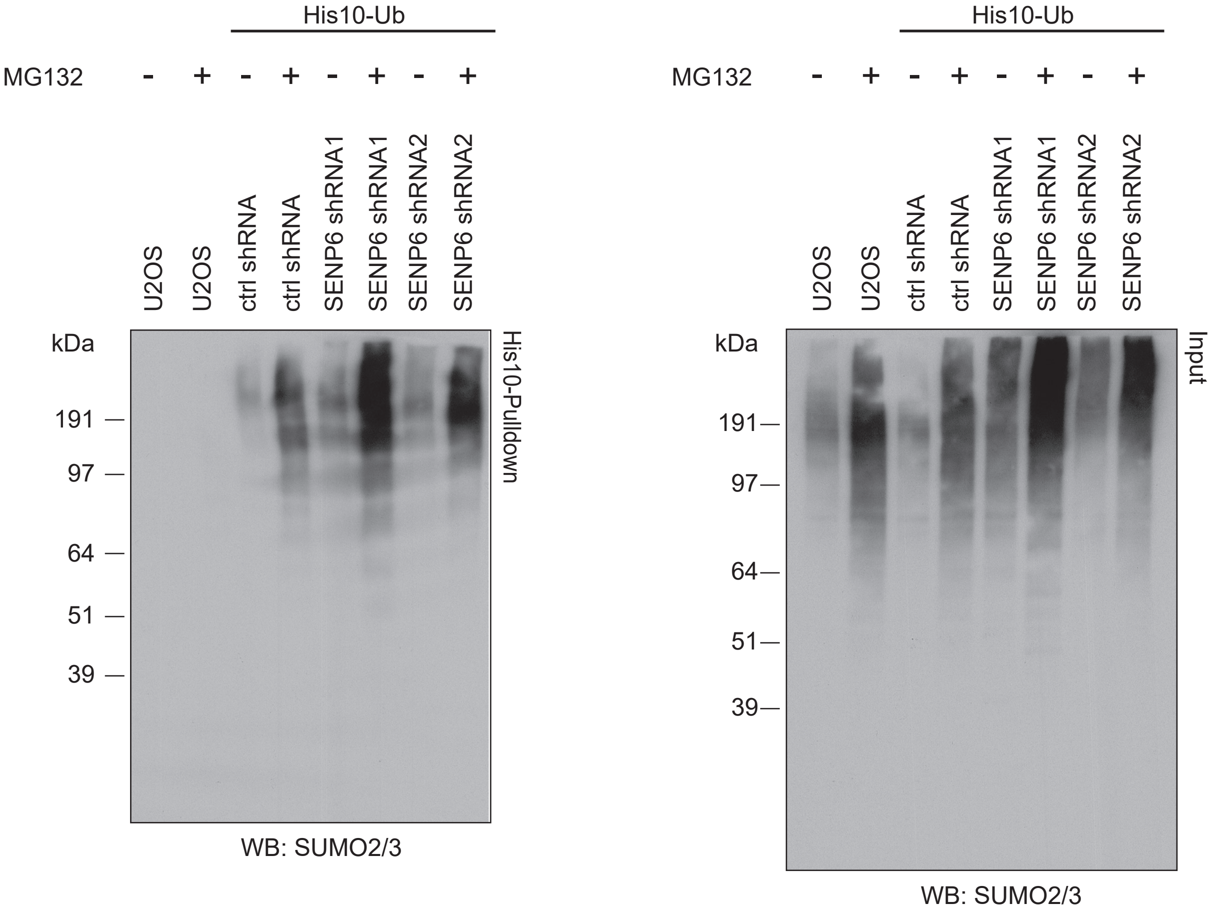

His10-Ub
MG132
- + - + - + - +
U2OS
U2OS
ctrl shRNA
ctrl shRNA
SENP6 shRNA1
SENP6 shRNA1
SENP6 shRNA2
SENP6 shRNA2
kDa
191
97
64
51
39
His10-Pulldown
WB: SUMO2/3
His10-Ub
MG132
- + - + - + - +
U2OS
U2OS
ctrl shRNA
ctrl shRNA
SENP6 shRNA1
SENP6 shRNA1
SENP6 shRNA2
SENP6 shRNA2
kDa
191
97
64
51
39
Input
WB: SUMO2/3

Supplementary Fig. 3 SENP6 knockdown increases SUMO and ubiquitin co-modified substrates.

a U2OS cells stably expressing His10-SUMO2 were infected with lentiviruses encoding shRNAs against SENP6 or a non-targeting control (ctrl) shRNA three days prior to lysis. Where indicated, cells were treated with 10 µM MG132 for 4 hours prior to lysis. Cells were lysed and SUMOylated proteins were enriched by means of Ni-NTA pulldown. Inputs and His10-purified samples were analysed by immunoblotting, using an antibody against ubiquitin. The observed increase of ubiquitin conjugates confirms efficient inhibition of the proteasome by MG132.

b U2OS cells stably expressing His10-ubiquitin were treated as in panel a. Input and His10-purified samples were analysed by immunoblotting, using an antibody against SUMO2/3. As expected, inhibition of the proteasome led to a global increase in SUMO conjugates. Source data are provided as a Source Data file.

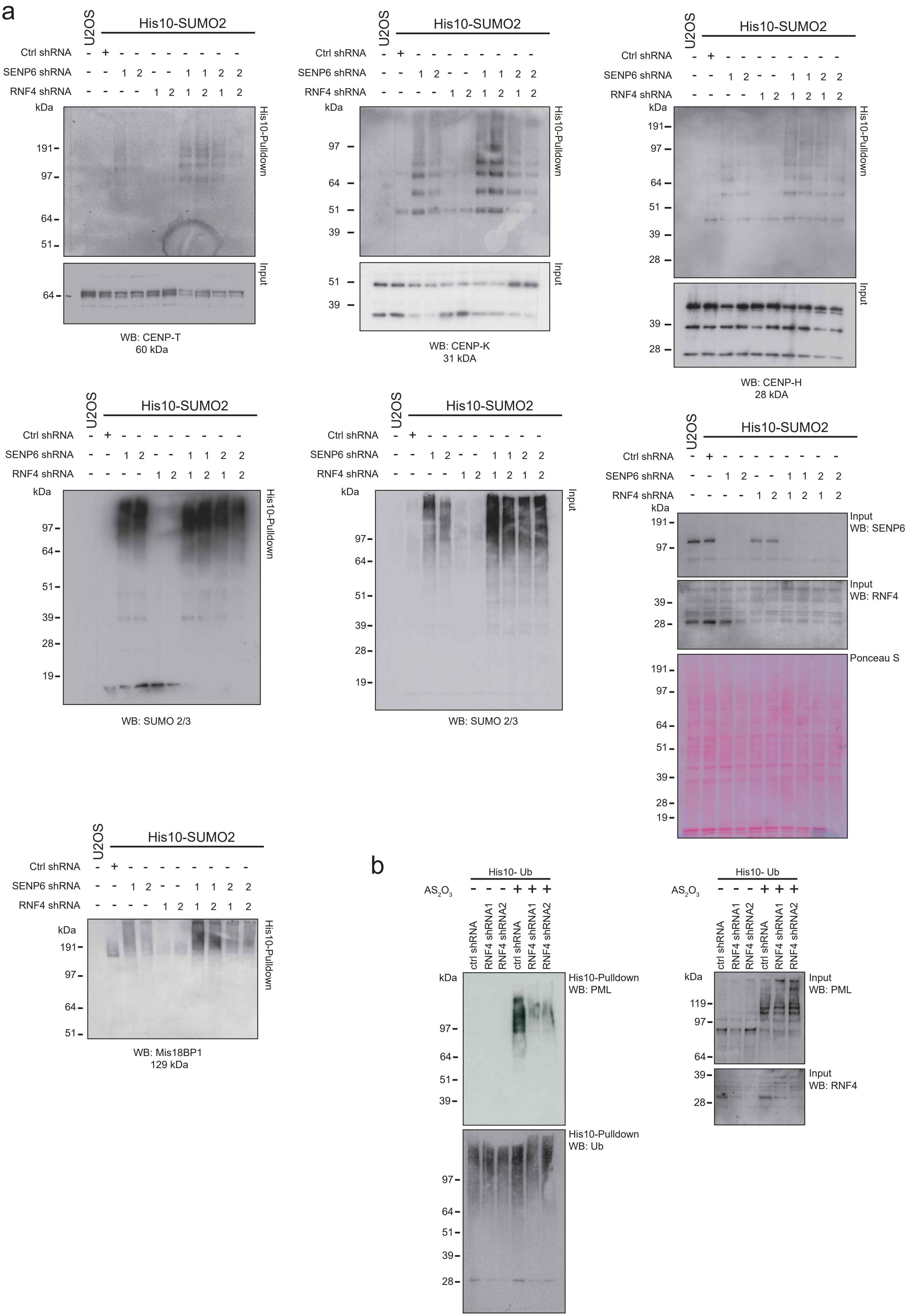
a
U2OS
His10-SUMO2
Ctrl shRNA
SENP6 shRNA
RNF4 shRNA
kDa
His10-Pulldown
Input
WB: CENP-T
60 kDa
WB: CENP-K
31 kDA
WB: CENP-H
28 kDA
WB: SUMO 2/3
WB: SUMO 2/3
Input
WB: SENP6
Input
WB: RNF4
Ponceau S
WB: Mis18BP1
129 kDa
b
His10- Ub
AS_2O_3
ctrl shRNA
RNF4 shRNA1
RNF4 shRNA2
His10-Pulldown
WB: PML
His10-Pulldown
WB: Ub
Input
WB: PML
Input
WB: RNF4

Supplementary Fig. 4 SUMOylated CCAN subunits are not stabilized upon RNF4 knockdown.

a U2OS cells stably expressing His10-SUMO2 were infected with lentiviruses encoding either one of the two shRNAs directed against SENP6 or against RNF4 or co-infected with a combination of one SENP6- and one RNF4-directed shRNA as indicated. Cells were lysed 3 days post infection and SUMOylated proteins were enriched by means of Ni-NTA pulldown. Inputs and His10-purified samples were analysed by immunoblotting, using antibodies against the indicated proteins. Compared to SENP6 depletion alone, co-depletion with RNF4 did not lead to a stabilization of SUMOylated CENP-T, CENP-K and CENP-H, but did lead to a stabilization of SUMOylated Mis18BP1.

b U2OS cells stably expressing His10-ubiquitin were infected with lentiviruses encoding shRNAs directed against RNF4 or a control shRNA (ctrl) and lysed two days post infection. Where indicated, cells were treated with 1µM arsenic trioxide (AS_2O_3) for four hours before lysis. Ubiquitinated proteins were enriched by means of Ni-NTA pulldown. Inputs and His10-purified samples were analysed by immunoblotting, using antibodies against the indicated proteins. PML ubiquitination is induced by AS_2O_3 in a RNF4-dependent manner, validating the shRNA-mediated RNF4 knockdown used. Source data are provided as a Source Data file.

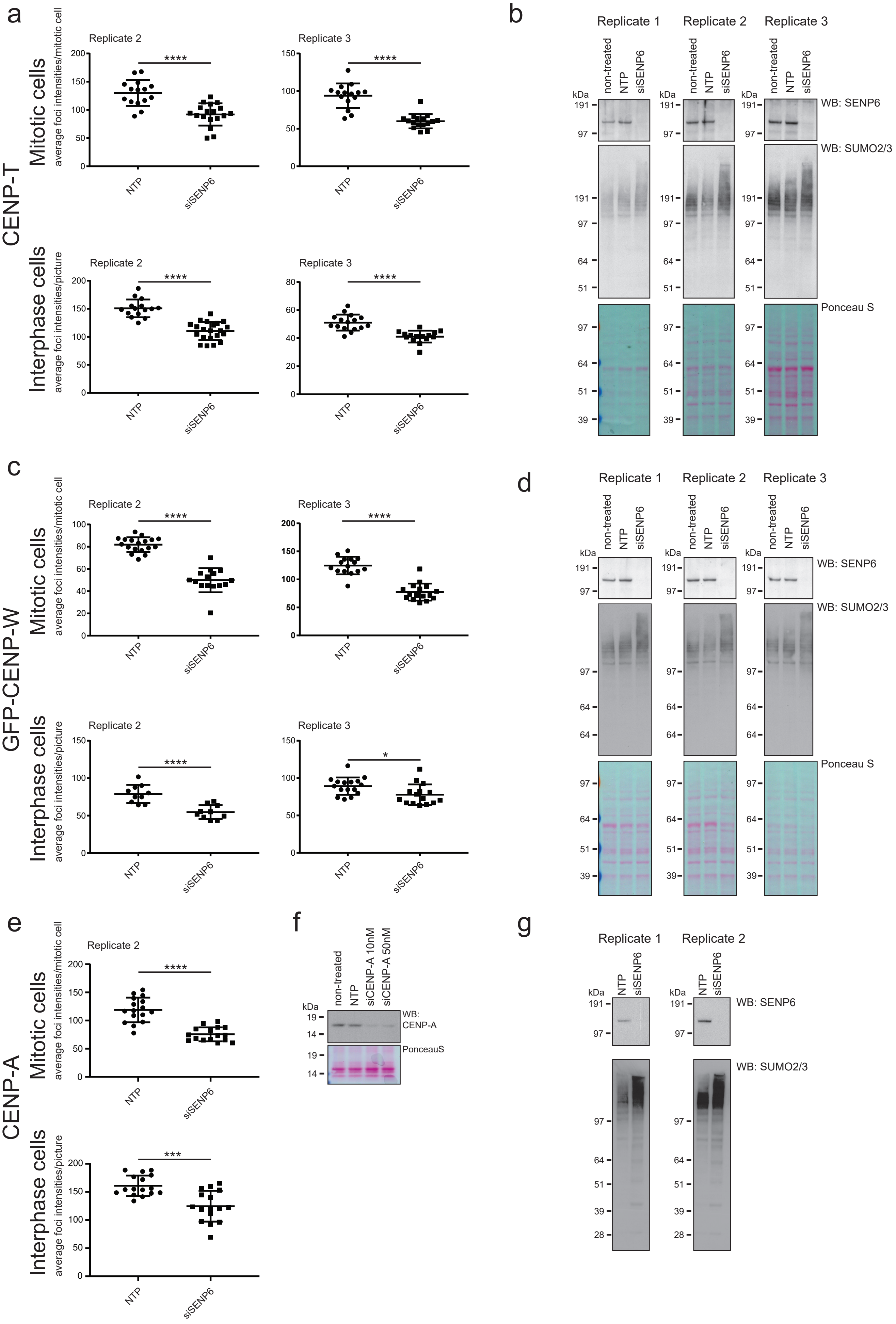
a
CENP-T
Mitotic cells
average foci intensities/mitotic cell
Replicate 2
Replicate 3
****
NTP
siSENP6
Interphase cells
average foci intensities/picture
b
Replicate 1
Replicate 2
Replicate 3
non-treated
NTP
siSENP6
kDa
WB: SENP6
WB: SUMO2/3
Ponceau S
c
GFP-CENP-W
*
d
e
CENP-A
***
f
siCENP-A 10nM
siCENP-A 50nM
WB:
CENP-A
PonceauS
g

Supplementary Fig. 5 The inner kinetochore proteins are less abundant at the centromere upon SENP6 knockdown.

a Quantifications of replicate 2 and 3 of CENP-T foci analysis shown in Fig. 6a. Scatterplots show quantification of the average CENP-T foci intensities per cell (mitotic cells) or per picture (interphase cells). A two-sided t-test was performed. ****p <0.0001. Replicate 2: n (NTP mitotic cells) = 15; n (siSENP6 mitotic cells) = 18; n (NTP interphase cells) = 15; n (siSENP6 interphase cells) = 21; Replicate 3: n (NTP mitotic cells) = 15; n (siSENP6 mitotic cells) = 16; n (NTP interphase cells) = 16; n (siSENP6 interphase cells) = 15.

b Immunoblot analysis of lysates from non-treated cells or cells treated with a pool of four siRNAs against SENP6 (siSENP6) or a pool of four non-targeting siRNAs (NTP), confirming efficient SENP6 knockdown, leading to high-molecular weight SUMOylated proteins. Cells were set up and treated in parallel to cells used in panel a and Fig. 6a. Loading was verified by Ponceau S staining.

c Quantifications of replicate 2 and 3 of CENP-W foci analysis shown in Fig. 6b. Scatterplots show quantification of the average CENP-W foci intensities per cell (mitotic cells) or per picture (interphase cells). A two-sided t-test was performed. ****p <0.0001, *p <0.05. Replicate 2: n (NTP mitotic cells) = 18; n (siSENP6 mitotic cells) = 15; n (NTP interphase cells) = 10; n (siSENP6 interphase cells) = 10; Replicate 3: n (NTP mitotic cells) = 15; n (siSENP6 mitotic cells) = 16; n (NTP interphase cells) = 16; n (siSENP6 interphase cells) = 15.

d Immunoblot analysis of lysates from non-treated cells or cells treated with a pool of four siRNAs against SENP6 (siSENP6) or a pool of four non-targeting siRNA (NTP), confirming efficient SENP6 knockdown leading to high-molecular weight SUMOylated proteins. Cells were set up and treated in parallel to cells used in panel c and Fig. 6b. Loading was verified by Ponceau S staining.

e Quantifications of replicate 2 of CENP-A foci analysis shown in Fig. 6c. Scatterplots show quantification of the average CENP-A foci intensities per cell (mitotic cells) or per picture

(interphase cells). A two-sided t-test was performed. ****p <0.0001. Replicate 2: n (NTP mitotic cells) = 16; n (siSENP6 mitotic cells) = 16; n (NTP interphase cells) = 16; n (siSENP6 interphase cells) = 16.

f U2OS cells were either left untreated or treated with a pool of four non-targeting siRNAs (NTP) or a pool of four siRNA targeting CENP-A (siCENP-A). Cells were lysed two days post transfection and lysates were analysed by immunoblotting with antibodies against CENP-A. CENP-A antibody signal is reduced in cells treated with siCENP-A, validating the specificity of the antibody.

g Immunoblot analysis of lysates from cells treated with a pool of four siRNAs against SENP6 (siSENP6) or a pool of four non-targeting siRNA (NTP), confirming efficient SENP6 knockdown leading to high-molecular weight SUMOylated proteins. Cells were set up and treated in parallel to cells used in panel e and Fig. 6c. Source data are provided as a Source Data file. All Error bars shown represent standard deviations.

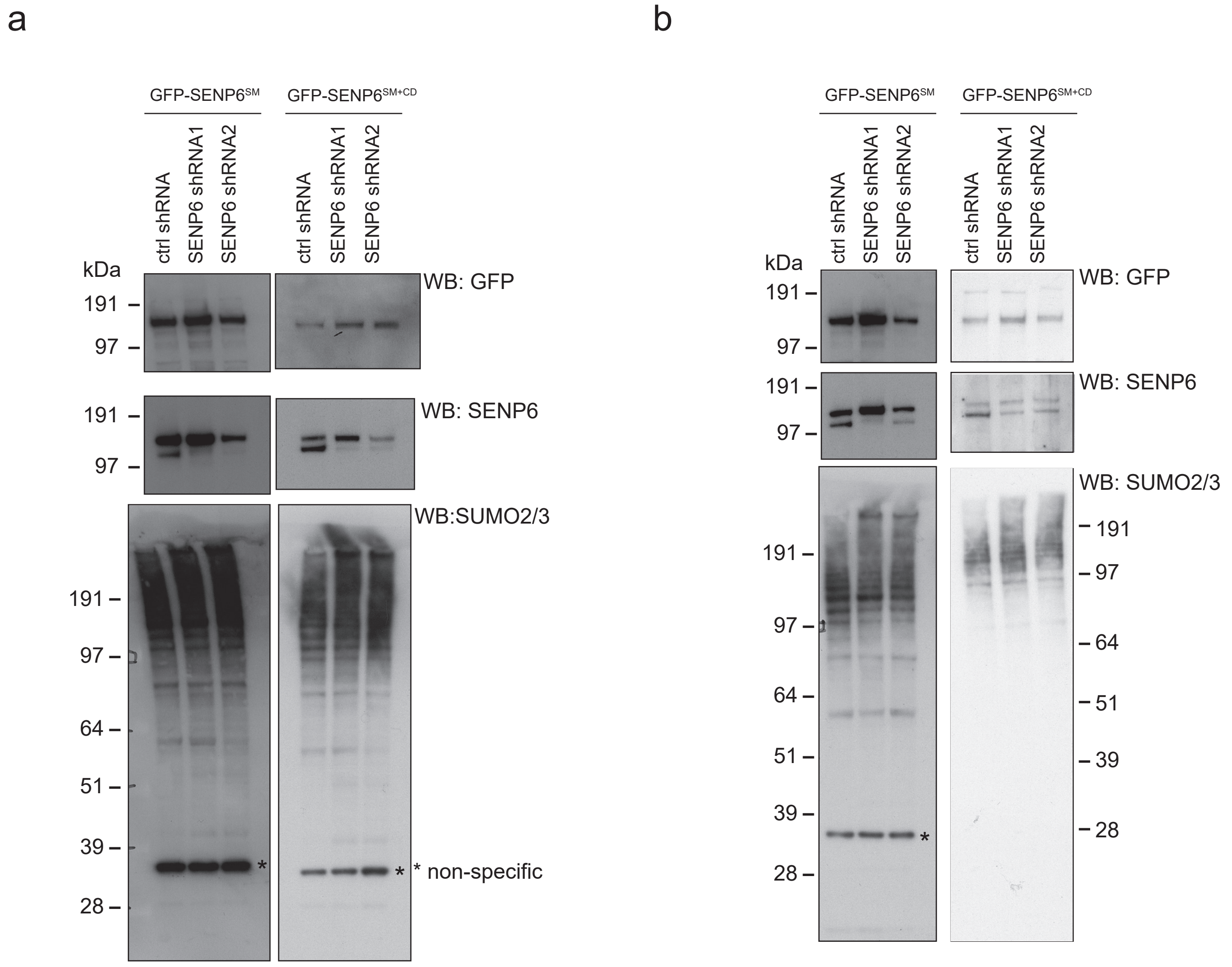
a
b
GFP-SENP6^SM
GFP-SENP6^SM+CD
ctrl shRNA
SENP6 shRNA1
SENP6 shRNA2
kDa
WB: GFP
WB: SENP6
WB:SUMO2/3
WB: SUMO2/3
191
97
64
51
39
28
* non-specific

Supplementary Fig. 6 Knockdown of SENP6 can be rescued by re-introduction of wild-type SENP6, but not by re-introduction of catalytic dead SENP6.

a and **b** U2OS cells stably expressing inducible shRNA-resistant (SM) GFP-tagged wild-type or catalytic dead (CD) SENP6 were established. Expression of these constructs was induced by Doxycycline for 24 hours prior to transduction with lentiviruses encoding SENP6 shRNAs. Medium was replaced one day post infection. The next day, cells were seeded in 12-well plates and grown overnight. Subsequently, cells were lysed and immunoblotting was performed using antibodies against GFP, SENP6 and SUMO2/3 to verify knockdown and rescue. * indicates a non-specific band. Results of the first replicate are shown in **a** and results of the second replicate are shown in **b**. Source data are provided as a Source Data file.

a

SIMa : (PILVM)-(ILVM)-X-(ILVM)-(DSE)(3)

SIMb : (PILVM)-(ILVM)-D-L-T

SIMr : (DSE)(3)-(ILVM)-X-(ILVMF)(2)

Protein	SIMa	SIMb	SIMr
CENP-C	No	No	932-938 EESVLLF
CENP-K	No	No	204-210 ESSVNLI
CENP-I	No	No	106-112 SEEIDIL
CENP-P	No	No	64-70 ESELSFL

b

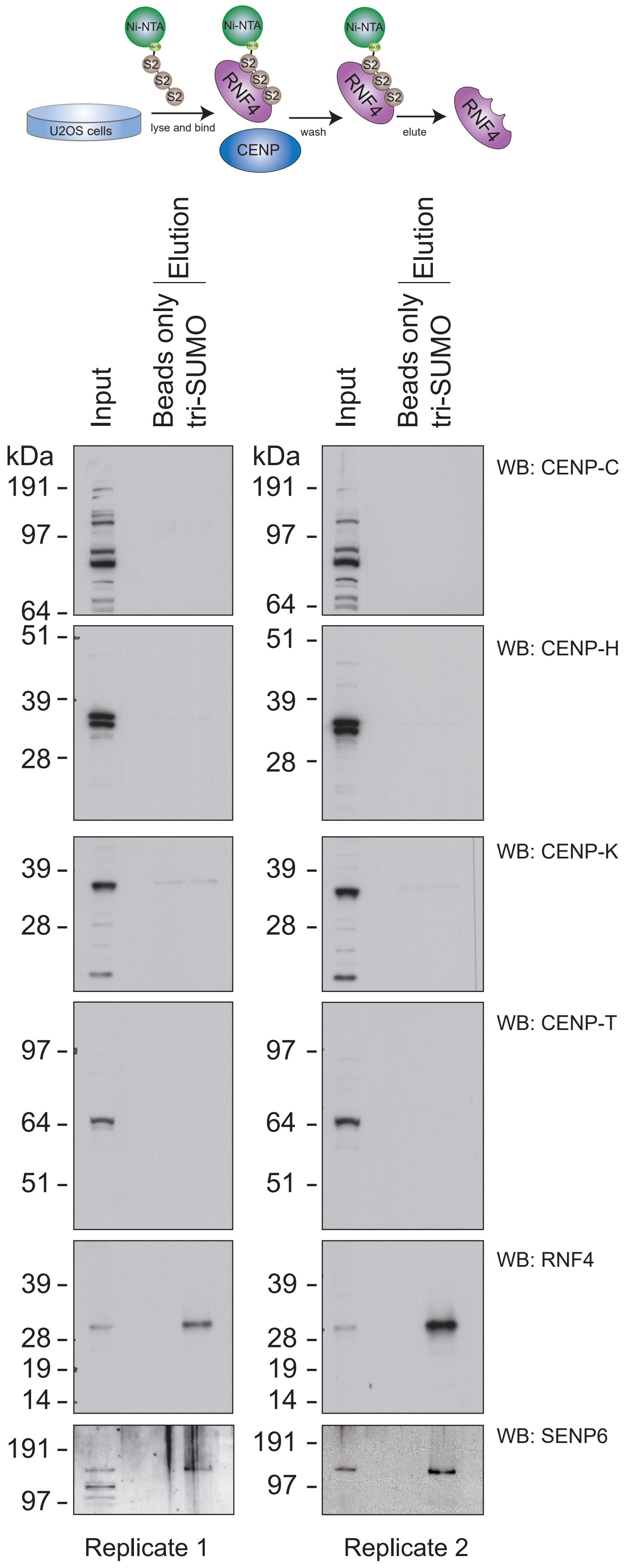

Supplementary Fig. 7 CCAN proteins are unable to bind SUMO trimer.

a Three consensus SUMO interaction motifs (SIMs) are defined by multiple hydrophobic residues (P,I,L,V or M), either followed or preceded by acidic residues (D or E) and residues which can be phosphorylated (S or T). The table shows the presence of these consensus SIM motifs within the 16 CCAN subunits. Only four CCAN subunits (CENP-C, -K, -I and -P) contain sequence-defined predicted SIMs. Subunits, location and sequences of predicted SIMs are listed.

b His10-SUMO2-trimer binding proteins were purified from U2OS lysates and subsequently analysed by immunoblotting using antibodies against the indicated proteins. Selected CENPs do not bind to recombinant SUMO-trimer. RNF4 and SENP6 were included as positive controls. Source data are provided as a Source Data file.

Supplementary Table 1

Antibodies

Name of antibody	Source	Catalogue number or reference	Dilution used
Mouse monoclonal anti-SUMO2/3	University of Iowa	8A2	1:500
Mouse monoclonal anti-Ubiquitilated proteins, clone FK2	Millipore	Cat# 04-263	1:1000
Mouse monoclonal anti-SENP6	Sigma-Aldrich	Cat# WHP0026054M1	WB 1:1000<br>IF 1:500
Mouse monoclonal anti-TopoIIα	BD Transduction Labs	611326	1:500
Mouse monoclonal anti-TopoIIβ	BD Transduction Labs	611492	1:500
Rabbit polyclonal anti-RNF4	NA	[1]	1:1000
Rabbit polyclonal anti-CENP-T	I. Cheeseman	[2]	WB 1:1000<br>IF 1:100
Rabbit polyclonal anti-CENP-I	P.T. Stukenberg	[3]	1:1000
Rabbit polyclonal anti-CENP-P/O	I. Cheeseman	[4]	1:1000
Rabbit polyclonal anti-CENP-C	W.C. Earnshaw	NA	1:1000
Rabbit polyclonal anti-CENP-H	I. Cheeseman	[2]	1:1000
Rabbit polyclonal anti-CENP-K	I. Cheeseman	[4]	1:1000
Rabbit polyclonal anti-CENP-B	W.C. Earnshaw	NA	1:1000
Mouse monoclonal anti-CENP-A	Abcam	Cat# ab13939	WB 1:1000<br>IF 1:100
Rabbit polyclonal anti-CENP-A	Cell Signaling Technology	Cat# 2186	IF 1:100
Rabbit polyclonal anti-KIF18A	Bethyl	Cat# A301-080A	1:1000
Rabbit monoclonal anti-KIF23	Abcam	Cat# ab174304	1:1000
Rabbit polyclonal anti-MIS18BP1	Bethyl	Cat# A302-825A	1:1000
Rabbit polyclonal anti-PML	Bethyl	Cat# A301-167A	1:2000
Rabbit polyclonal anti-GFP	Novus Biologicals	Cat# NB600-308	IF and WB 1:500
Rabbit monoclonal anti-β-tubulin	Cell Signaling Technology	Cat# 2128S	1:2000
Rabbit polyclonal anti-Histone H4	Abcam	Cat# ab10158-100	1:2000
Sheep polyclonal anti-SENP7	R.T. Hay	[5]	1:3000

Supplementary Table 2

Oligonucleotides and plasmid DNA

Sequence or Name	Source and purpose	Catalogue number or ID
SMART pool ON-TARGETplus SENP6 siRNA	Dharmacon	L-006044-00-0010
SMART pool ON-TARGET CENPA siRNA	Dharmacon	L-003249-00-0005
SMART pool ON-TARGET siRNA non-targeting	Dharmacon	D-001810-10-05
GGTGTATATGTATTGCAGTAAGTAGAGAGCTTTTTTGAGAATCC	This study, To generate SENP6 catalytically dead mutant	SENP6_C1030S_FW
GGATTCTCAAAAAAGCTCTCTACTTACTGCAATACATATACACC	This study, To generate SENP6 catalytically dead mutant	SENP6_C1030S_REV
GGAGGACAGAAGTCACAAAATACCGGCTTGACCACTAAAAAGTTTTATGGCAACAATGTGG	This study, To generate silent mutations in SENP6	SENP6_silent1024_FW
CCACATTGTTGCCATAAAACTTTTTAGTGGTCAAGCCGGTATTTTGTGACTTCTGTCCTCC	This study, To generate silent mutations in SENP6	SENP6_silent1024_REV
CCAGTGATGATGATGATGACAACGATAGGACCAATAGGAGGGAGAGCATATCTCCTCAGCC	This study, To generate silent mutations in SENP6	SENP6_silent1023_FW
GGCTGAGGAGATATGCTCTCCCTCCTATTGGTCCTATCGTTGTCATCATCATCATCACTGG	This study, To generate silent mutations in SENP6	SENP6_silent1023_REV
CAT CGA CGT GTT CCG GCA GCA GAC GGG AG	This study, To generate Flag-SUMO2-Q87R	2183_FW
CTC CCG TCT GCT GCC GGA ACA CGT CGA TG	This study, To generate Flag-SUMO2-Q87R	2183_REV
shRNA SENP6 #1-GACAGAACTAACAGAAGAGAA	Sigma-Aldrich Mission® shRNA library	TRC – 004103
shRNA SENP6 #2 – CACAGGATTAACAACCAAGAA	Sigma-Aldrich Mission® shRNA library	TRC – 004104

shRNA RNF4 #1 – CATACTCCCAGAAACGCCAGG	Sigma-Aldrich Mission® shRNA library	TRC – 272668
shRNA RNF4 #2 – CATACTCCCAGAAACGCCAGG	Sigma-Aldrich Mission® shRNA library	TRC – 284821
Non-targeting shRNA	Sigma-Aldrich Mission® shRNA library	SHC002
pBabe-CENP-W-GFP	This study	
pHIS-TEV30a:His10-ΔN11-SUMO2-trimer	[6]	
pCSF107mT-CENP-T-FLAG	This study	
pCSF107mT-CENP-T-3HA	This study	
pCW57.1-GFP-SENP6-kd-resistant w.t.	This study	
pCW57.1-GFP-SENP6-kd-resistant cat. dead	This study	
pCW57.1-GFP-SENP6 w.t.	This study	
pET28a-N-His-SENP6 (Catalytic domain)	Addgene	#16359
pGEX-2T-SUMO2 K0	[6]	
pGEX-2T GST-thrombin-flag-preScission-SUMO2 Q87R	This study	
pGEX-4T-SAE1/SAE2	[7]	
pGEX-2T-UBC9	[7]	
pGEX-2T-SUMO2	[7]	

Supplementary references

1. Vyas, R. *et al.* RNF4 is required for DNA double-strand break repair in vivo. *Cell Death Differ* **20**, 490-502 (2013).
2. Gascoigne, K.E. *et al.* Induced ectopic kinetochore assembly bypasses the requirement for CENP-A nucleosomes. *Cell* **145**, 410-422 (2011).
3. Matson, D.R., Demirel, P.B., Stukenberg, P.T. & Burke, D.J. A conserved role for COMA/CENP-H/I/N kinetochore proteins in the spindle checkpoint. *Genes Dev* **26**, 542-547 (2012).
4. McKinley, K.L. *et al.* The CENP-L-N Complex Forms a Critical Node in an Integrated Meshwork of Interactions at the Centromere-Kinetochore Interface. *Mol Cell* **60**, 886-898 (2015).
5. Shen, L.N., Geoffroy, M.C., Jaffray, E.G. & Hay, R.T. Characterization of SENP7, a SUMO-2/3-specific isopeptidase. *Biochem.J.* **421**, 223-230 (2009).
6. Eifler, K. *et al.* SUMO targets the APC/C to regulate transition from metaphase to anaphase. *Nat Commun* **9**, 1119 (2018).
7. Tatham, M.H. *et al.* Polymeric chains of SUMO-2 and SUMO-3 are conjugated to protein substrates by SAE1/SAE2 and Ubc9. *J.Biol.Chem.* **276**, 35368-35374 (2001).
