# Supplementary material for: The poly-SUMO2/3 protease SENP6 enables assembly of the constitutive centromere-associated network by group deSUMOylation
Source: Nat Commun. 2019 Sep 4;10:3987. doi: 10.1038/s41467-019-11773-x (PMC6726658; doi:10.1038/s41467-019-11773-x)
Supplement: Supplementary file 3 — Description of Additional Supplementary Files [file 41467_2019_11773_MOESM3_ESM.docx]

**Description of Supplementary Files**

**File Name:** **Supplementary Data 1**

**Description:** Complete list of identified protein groups including statistics, Related to Fig. 2

**File Name:** **Supplementary Data 2**

**Description:** List of putative SENP6 regulated SUMOylated proteins, Related to Fig. 3

**File Name: Supplementary Data 3**

**Description:** List of chromatin-associated proteins, Related to Fig. 9
