# Supplementary material for: The poly-SUMO2/3 protease SENP6 enables assembly of the constitutive centromere-associated network by group deSUMOylation
Source: Nat Commun. 2019 Sep 4;10:3987. doi: 10.1038/s41467-019-11773-x (PMC6726658; doi:10.1038/s41467-019-11773-x)
Supplement: Supplementary file 7 — Reporting Summary [file 41467_2019_11773_MOESM7_ESM.pdf]

nature research

Corresponding author(s): Alfred Vertegaal

Last updated by author(s): Jul 22, 2019

Reporting Summary

Nature Research wishes to improve the reproducibility of the work that we publish. This form provides structure for consistency and transparency in reporting. For further information on Nature Research policies, see Authors & Referees and the Editorial Policy Checklist.

Statistics

For all statistical analyses, confirm that the following items are present in the figure legend, table legend, main text, or Methods section.

n/a	Confirmed	
☐	☒	The exact sample size (*n*) for each experimental group/condition, given as a discrete number and unit of measurement
☐	☒	A statement on whether measurements were taken from distinct samples or whether the same sample was measured repeatedly
☐	☒	The statistical test(s) used AND whether they are one- or two-sided<br>*Only common tests should be described solely by name; describe more complex techniques in the Methods section.*
☒	☐	A description of all covariates tested
☐	☒	A description of any assumptions or corrections, such as tests of normality and adjustment for multiple comparisons
☐	☒	A full description of the statistical parameters including central tendency (e.g. means) or other basic estimates (e.g. regression coefficient) AND variation (e.g. standard deviation) or associated estimates of uncertainty (e.g. confidence intervals)
☐	☒	For null hypothesis testing, the test statistic (e.g. *F*, *t*, *r*) with confidence intervals, effect sizes, degrees of freedom and *P* value noted<br>*Give P values as exact values whenever suitable.*
☒	☐	For Bayesian analysis, information on the choice of priors and Markov chain Monte Carlo settings
☒	☐	For hierarchical and complex designs, identification of the appropriate level for tests and full reporting of outcomes
☒	☐	Estimates of effect sizes (e.g. Cohen's *d*, Pearson's *r*), indicating how they were calculated

Our web collection on statistics for biologists contains articles on many of the points above.

Software and code

Policy information about availability of computer code

Data collection	BD FACS DIVA software version 8.0 www.bdbiosciences.com<br>Leica Application Suite X www.leica-microsystems.com<br>Xcalibur version 3.1 www.thermofisher.com
Data analysis	Cytoscape version 3.6.1 www.cytoscape.org<br>MaxQuant version 1.5.3.30 \www.coxdocs.org/doku.php?id=maxquant:start<br>Perseus 1.5.0.31 \www.coxdocs.org/doku.php?id=perseus:start<br>Graphpad Prism7 www.graphpad.com<br>ImageJ2 from Fiji www.fiji.sc<br>FlowJo v.10 www.flowjo.com<br>STRING database version 10.5 https://string-db.org

For manuscripts utilizing custom algorithms or software that are central to the research but not yet described in published literature, software must be made available to editors/reviewers. We strongly encourage code deposition in a community repository (e.g. GitHub). See the Nature Research guidelines for submitting code & software for further information.

Data

Policy information about availability of data

All manuscripts must include a data availability statement. This statement should provide the following information, where applicable:

- Accession codes, unique identifiers, or web links for publicly available datasets
- A list of figures that have associated raw data
- A description of any restrictions on data availability

The mass spectrometry proteomics data have been deposited to the ProteomeXchange Consortium via the PRIDE partner repository with the dataset identifier PXD011963.

Field-specific reporting

Please select the one below that is the best fit for your research. If you are not sure, read the appropriate sections before making your selection.

☒ Life sciences ☐ Behavioural & social sciences ☐ Ecological, evolutionary & environmental sciences

For a reference copy of the document with all sections, see nature.com/documents/nr-reporting-summary-flat.pdf

Life sciences study design

All studies must disclose on these points even when the disclosure is negative.

Sample size	sample sizes were chosen according to common practice in the field.
Data exclusions	No data were excluded from the analyses.
Replication	Experiments were independently repeated, yielding reproducible data. The exact sample sizes (n) are specified in the legends.
Randomization	not applicable.
Blinding	blinding was not applicable.

Reporting for specific materials, systems and methods

We require information from authors about some types of materials, experimental systems and methods used in many studies. Here, indicate whether each material, system or method listed is relevant to your study. If you are not sure if a list item applies to your research, read the appropriate section before selecting a response.

Materials & experimental systems

n/a	Involved in the study	
☐	☒	Antibodies
☐	☒	Eukaryotic cell lines
☒	☐	Palaeontology
☒	☐	Animals and other organisms
☒	☐	Human research participants
☒	☐	Clinical data

Methods

n/a	Involved in the study	
☒	☐	ChIP-seq
☐	☒	Flow cytometry
☒	☐	MRI-based neuroimaging

Antibodies

Antibodies used

Mouse monoclonal anti-SUMO2/3 University of Iowa 8A2
Mouse monoclonal anti-Ubiquitilated proteins, clone FK2 Millipore Cat# 04-263
Mouse monoclonal anti-SENP6 Sigma-Aldrich Cat# WHP0026054M1
Mouse monoclonal anti-TopoIIα BD Transduction Labs Cat# 611326
Mouse monoclonal anti-TopoIIβ BD Transduction Labs Cat# 611492
Rabbit polyclonal anti-RNF4 NA (Vyas et al., 2013)
Rabbit polyclonal anti-CENP-T I. Cheeseman (Gascoigne et al., 2011)
Rabbit polyclonal anti-CENP-I P.T. Stukenberg (Matson et al., 2012)
Rabbit polyclonal anti-CENP-P/O I. Cheeseman (McKinley et al., 2015)
Rabbit polyclonal anti-CENP-C W.C. Earnshaw NA
Rabbit polyclonal anti-CENP-H I. Cheeseman (Gascoigne et al., 2011)
Rabbit polyclonal anti-CENP-K I. Cheeseman (McKinley et al., 2015)
Rabbit polyclonal anti-CENP-B W.C. Earnshaw NA
Rabbit polyclonal anti-CENP-A Abcam Cat# ab13939
Rabbit polyclonal anti-CENP-A Cell Signaling Technology Cat# 2186
Rabbit polyclonal anti-KIF18A Bethyl Cat# A301-080A
Rabbit monoclonal anti-KIF23 Abcam Cat# ab174304
Rabbit polyclonal anti-MIS18BP1 Bethyl Cat# A302-825A
Rabbit polyclonal anti-PML BethylA Cat# A301-167A
Rabbit polyclonal anti-GFP Novus Biologicals Cat# NB600-308
Rabbit monoclonal anti-β-tubulin Cell Signaling technology Cat# 2128S
Rabbit polyclonal anti-Histone H4 Abcam Cat# ab10158-100
Sheep polyclonal anti-SENP7 R.T. Hay (Shen et al. 2009)

Validation

The antibodies described above are validated on the websites of the manufacturer and in the cited articles

Eukaryotic cell lines

Policy information about cell lines

Cell line source(s)	U2OS were derived from the ATCC<br>HeLa cells were derived from the EMBL
Authentication	Cell lines have been authenticated via STR profiling using 10 different markers
Mycoplasma contamination	Cell lines have been tested to be free of mycoplasma
Commonly misidentified lines<br>(See ICLAC register)	not applicable

Flow Cytometry

Plots

Confirm that:

☒ The axis labels state the marker and fluorochrome used (e.g. CD4-FITC).

☒ The axis scales are clearly visible. Include numbers along axes only for bottom left plot of group (a 'group' is an analysis of identical markers).

☒ All plots are contour plots with outliers or pseudocolor plots.

☒ A numerical value for number of cells or percentage (with statistics) is provided.

Methodology

Sample preparation	HeLa cells were harvested by trypsinization washed once in PBS and resuspended in 1 ml of PBS. 4 ml of 100 % ethanol was added and the cells were fixed at 4ºC overnight. On the day of flow cytometry analysis, the cells were first centrifuged at 500 xg for 2 minutes, the supernatant was removed and the cells were washed with PBS and 2 % calf serum. Then, the cells were pelleted again and resuspended in 500 µl of PBS complemented with 2 % calf serum, 25 µg/ml propidium iodide (Sigma-Aldrich, P4170) and 100 µg/ml RNAse A (Sigma-Aldrich, R6513).
Instrument	BD LSRII system (BD Biosciences Clontech)
Software	Cell cycle analysis was performed with FlowJo version 10 software using the Watson univariate model.
Cell population abundance	The cell population consisted only of HeLa cells.
Gating strategy	The gating strategy was the inclusion of all single cells in our cell cycle analysis. The exact gating boundaries are provided in Supplementary Figure 2.

☒ Tick this box to confirm that a figure exemplifying the gating strategy is provided in the Supplementary Information.
